# The Sol platform: Integrating computation and ML/AI with structural data for research and drug discovery

**DOI:** 10.1063/4.0001057

**Published:** 2025-10-27

**Authors:** Seth F. Harris

**Affiliations:** Genentech, South San Francisco, CA, USA

## Abstract

We have created a platform to provide an integrated computational environment for structural biology-based research and drug discovery. By amassing key features of structures through pre-computed annotations we are able to achieve two-fold benefits. First, expert or non-expert users alike engaged in "traditional" target-focused discovery have instant access to an enriched data context for their structures of interest, navigable via an interactive visual interface that seamlessly projects data between the 2D and 3D molecular environments. Second, programmatic access allows these millions of pre-computed features to be efficiently provisioned for the development of new machine learning models and algorithms, and in a virtuous loop, those models are readily made available to end users for additional insights and shaping. I present a brief overview of the capabilities of the platform, and subsequently more detailed examples, particularly around binding pockets and interfaces, of how we accelerate our target assessment and machine learning-based hypothesis generation on protein and ligand interactions at scale, blending protein structure predictions and design in both large and small molecule drug discovery.

